# Suhaim-Turayki Technique for the Decompression of Odontogenic Keratocysts: A Case Series

**DOI:** 10.12688/f1000research.163210.1

**Published:** 2025-05-20

**Authors:** Faisal Bin Suhaim, Haitham Bin Turayki, Muslat A Bin Rubaia’an, Munira Alshahrani, Rana Alshagroud, Ra'ed Al-Sadhan

**Affiliations:** 1 Oral and Maxillofacial Surgery Department, King Saud Medical City, Riyadh, Riyadh Province, Saudi Arabia; 2 Oral and Maxillofacial Surgery Department, King Khalid University, Abha, Aseer Province, Saudi Arabia; 3 Oral Medicine and Diagnostic Sciences, King Saud University, Riyadh, Riyadh Province, Saudi Arabia

**Keywords:** Odontogenic cyst, conservative, drainage, resolution, bone regeneration

## Abstract

**Purpose:**

This report focuses on the efficacy of the Suhaim-Turayki Technique, a modified decompression procedure using dual drainage openings, in the treatment of odontogenic keratocysts (OKCs).

**Case Presentation:**

We present five cases (four females, one male; age range: 25–31 years) of patients treated with our novel technique for OKCs. The cysts were located primarily in the left mandible, except for one case in the anterior mandible. Two patients underwent the procedure under general anesthesia, while three were treated with local anesthesia. The cyst dimensions ranged from 5.2 to 7.3 cm in width and 1.7 to 5.8 cm in height. The procedure involved inserting a perforated Foley catheter drain (size 15 or 16) into the cystic cavity, with dual openings connected to the oral cavity for continuous drainage. Patients received weekly irrigation of the cavity, with subsequent home irrigation instructions. Follow-up intervals ranged from 6 to 12 months. Complete resolution was achieved in all cases, either with decompression alone or with an additional enucleation procedure for cyst remnants in two cases. At discharge, radiographs confirmed full bony regeneration and the absence of recurrence.

**Conclusion:**

The Suhaim-Turayki Technique proved to be a timely and effective method for managing OKCs, with complete cyst resolution and no recurrences observed during extended follow-up. This modified decompression technique is a promising alternative for patients where more invasive treatment options are not suitable or preferred.

**Abbreviations**
OKC: Odontogenic Keratocyst
OMFS: Oral and Maxillofacial Surgery
F: Female
M: Male
W: Width
H: Hight
GA: General Anesthesia
LA: Local Anesthesia
CM: Centimeter
OPG: Orthopantomogram
CBCT: Cone Beam Computed Tomography
WHO: World Health Organization

## Introduction

Odontogenic keratocysts (OKCs) are abnormal cysts with a high probability of recurrence. ^1^ Several studies have suggested that OKCs can be classified as tumoral lesions due to their aggressive and destructive nature. ^2, 3^ Odontogenic keratocysts have been observed in 11 percent of odontogenic cyst cases. ^4^ They are most commonly observed in patients between the ages of 20 and 30. ^1^ The prevalence of OKCs in women peaks approximately 10 years earlier than it does in men. The reoccurrence rate of OKCs ranges from 10–60%, ^1^ and OKCs have been recognized as a common abnormality of dental origin. ^5^

From a clinical perspective, OKCs commonly occur in the posterior region of the mandible. ^6^ Odontogenic keratocysts can exhibit distinct unilocular or multilocular radiolucency that is either connected to or unconnected from an unerupted tooth. ^6^

Several treatment methods for OKCs have been documented, and those methods are associated with varying rates of recurrence. Enucleation, cryotherapy-assisted enucleation, marsupialization, decompression, and resection are common treatment methods. ^7^ Marsupialization and decompression are distinct surgical techniques. These approaches are intended to reduce the size of large OKCs prior to removal or to completely resolve the lesion. ^8^ The primary benefit of conservative management is the preservation of crucial anatomical structures, such as the inferior alveolar nerve, and the prevention of potential deformities that may result from other aggressive approaches. ^9– 11^ Some researchers contend that, given the elevated rates of recurrence of OKCs, it is advisable to pursue more assertive treatment options. ^1, 4, 12^ Nevertheless, resection can result in a range of additional issues, such as dental and bone changes, numbness in the lower lip, and difficulties chewing. ^13, 14^ Therefore, it is advisable to prioritize conservative treatment over alternative methods for patients with OKCs.

Decompression is a less-invasive procedure compared with marsupialization, enucleation, curettage, and resection because it involves creating a smaller opening in the bone. During the decompression procedure, a drain is inserted into the lesion to create a constant connection with the cystic cavity. Reducing intracystic pressure can lead to the development of new bone tissue. ^15^ Pogrel and Jordan noted that the terms “decompression” and “marsupialization” have used interchangeably in the literature. However, these terms refer to distinct approaches. ^8^

This paper highlights several cases of OKCs successfully effectively treated using the Suhaim-Turayki Technique, a modified decompression technique that employs two openings drains. Our investigation emphasizes the benefits of this modified technique while also providing an overview of its limitations.

## Case presentation

Four female and one male patient with cysts of odontogenic origin were treated by decompression at the Department of Oral and Maxillofacial Surgery (OMFS) at King Saud Medical City in Riyadh, Saudi Arabia ( Table 1).

**Table 1.** Description of clinical cases.

Gender	Age	Diagnosis	Location	Size WxH in cm	Anesthesia	Drain Use in Weeks	Complications				Outcomes		Follow-Up Duration After Cyst Resolution in Weeks
							Discomfort	Drain Dislodgment	Infection	Paresthesia	Bony Infill	Required 2nd Intervention	
F	25	OKC	Left Mandible	6.2x5.8	GA	58		X			X		128
M	31	OKC	Anterior Mandible	7.3x3.8	LA	56					X		384
F	29	OKC	Left Mandible	5.2x2.9	LA	32					X		116
F	30	OKC	Left Mandible	5.6x1.7	GA	28	X				X	Enucleation	92
F	28	OKC	Left Mandible	5.6x2.0	LA	25	x			x	x	Enucleation	73

**Notes: Abbreviations:** F, Female **;** M, Male **;** OKC, Odontogenic keratocyst **;** W, Width **;** H, Hight; GA, General anesthesia **;** LA, Local anesthesia **;** CM, Centimeter.

The patients ranged in age from 25–31 years. Most of the patients’ cysts were located in the left mandible; one cyst was located in the anterior mandible. Two patients were treated under general anesthesia, and three were treated under local anesthesia. The widths and heights of the cysts, measured on the orthopantomographs, varied from 5.2–7.3 cm and 1.7–5.8 cm, respectively. Four patients presented swelling and pain, and one patient had a history of altered sensation. Four cysts were asymptomatic. For each patient, we recorded a thorough medical history.

We used a novel technique in which a perforated drain, size 15 or 16 Foley’s catheter, was inserted into the cystic lesion and communicated to the oral cavity via two openings. The tube length was selected to match the distance between the anterior and posterior walls of the cyst and the oral mucosa. After the initial surgery, patients attended weekly follow-up visits for saline irrigation of the cavity. A few weeks later, they were given a syringe to continue irrigation at home, with follow-up appointments scheduled at intervals of 6 to 12 months.

Most cases in this series were managed solely through decompression. In two instances, a second surgical procedure was necessary to enucleate the residual cystic lining. All patients were monitored until complete cyst resolution was confirmed both clinically and radiographically.

Complete resolution was observed in cysts managed with simple surgical decompression, as well as in those requiring a second procedure to enucleate the downsized cyst.

At the time of discharge, all cysts had either fully externalized or undergone complete involution, resulting in intact mucosal coverage. Final radiographic evaluation confirmed complete bony infill. Following the resolution of the cysts, the patients were followed up at intervals ranging from approximately 1.4 to 7.4 years to monitor for long-term outcomes and potential recurrence.

### Protocol

A diagnosis of OKC is typically made based on radiological findings. When reviewing a patient’s medical history, it is important to investigate any prior treatment or reoccurrence of jaw lesions. A comprehensive clinical examination should be performed as well that specifically includes palpation to identify any possible swelling. It is necessary to assess the vitality and restorability of all teeth involved. An initial orthopantomogram (OPG) can offer an initial assessment of a lesion.

That initial OPG is particularly beneficial for subsequent follow-up. However, a comprehensive assessment of the actual size of the lesion (measured along the major axis) can only be obtained from a three-dimensional examination. A biopsy is necessary prior to making any further treatment decisions; only an anatomopathological examination can yield a definitive diagnosis.

Not all patients are suitable for decompression. The patient must be willing to adhere to a strict oral hygiene regimen and perform specific flushing procedures once or twice a day for several months.

**Figure 1.**
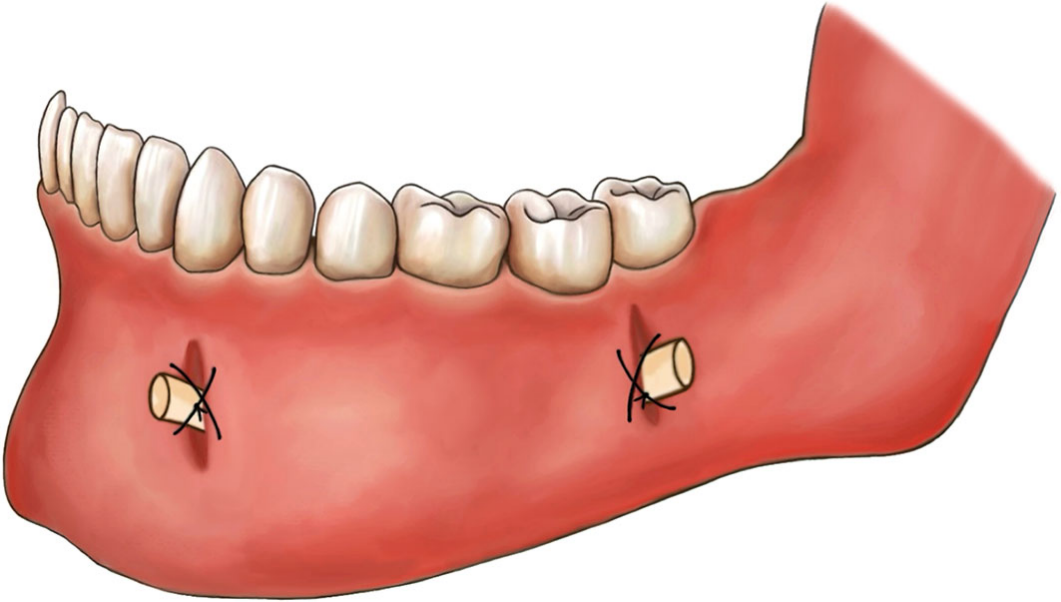
Suhaim-Turayki Technique: A modified decompression technique employing a drain with two openings.

The procedural instructions involved in decompression are as follows:

− Antisepsis of tissues
− Local or general anesthesia
− Extraction of any offending teeth
− Completion of a stab incision of the mucosa and the periosteum, beginning at the cyst and extending to its most anterior extension
− Elevation of the mucoperiosteal flap
− Osteotomy on the thinned bone using hemostatic forceps or rotary round bur (if necessary)
− Excision of the cystic membrane
− Transmission of the sample to the anatomical pathology container filled with 10% buffered formalin
− Repetition of the above steps for the most superior posterior extension, without excision
− Completion of multiple perforations on a size 15 or 16 Foley’s catheter drain manually with scissors and then insertion of the drain into the cyst cavity from the posterior window and advancement of it to the anterior window
− Stitching of the two drain openings to the surrounding mucosa using silk sutures ( Figure 1)

The required operating time is roughly 45–60 minutes. The surgeon has to make sure the patient can perform the rinse protocol correctly. Conducting a demonstration in front of a mirror can be useful for educating patients.

Periodic radiological and clinical evaluations are performed every two months to monitor the advancement of bony infill. The position of the drain is also confirmed. Upon radiological follow-up, new bone formation can be observed along the periphery of the cystic wall due to the decrease in intracystic pressure. The length of time required for decompression until resolution may vary based on the initial size of the lesion and the pace of reduction; it typically ranges from 6–14 months.

### Case 1

**Figure 2.**
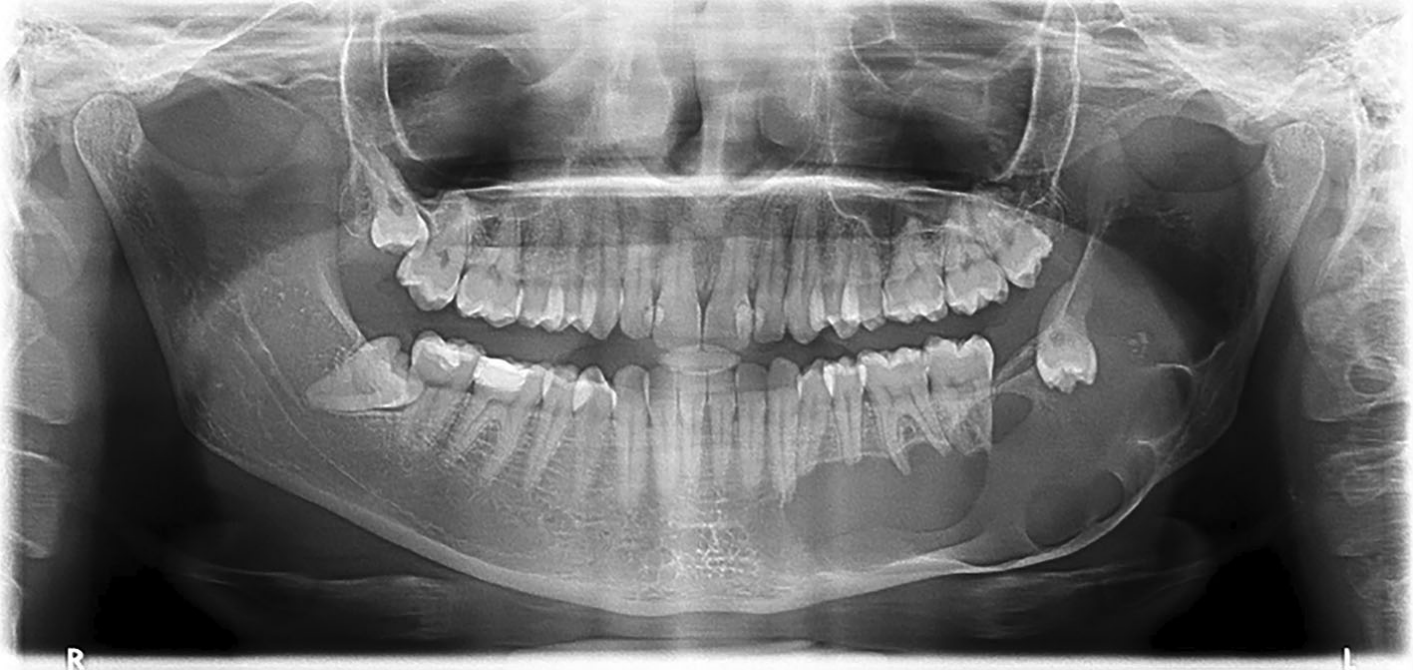
Case 1 pre-operative OPG, showing a large radiolucent lesion in the left mandible.

A 25-year-old female with no known medical conditions or allergies visited our outpatient department following the incidental discovery of a large left mandibular lesion upon her visit to a private clinic. Three weeks prior, she had noticed an asymptomatic swelling on the left side of her jaw. A physical examination revealed left facial swelling that caused noticeable asymmetry, with buccal obliteration observed intraorally. Clinical palpation revealed crepitation, but no paresthesia was noted. Her maximum mouth opening was normal, and an endodontic evaluation confirmed that all involved teeth were vital. An OPG revealed a cystic lesion measuring 6.2 by 5.8 cm in the patient’s left mandible ( Figure 2).

**Figure 3.**
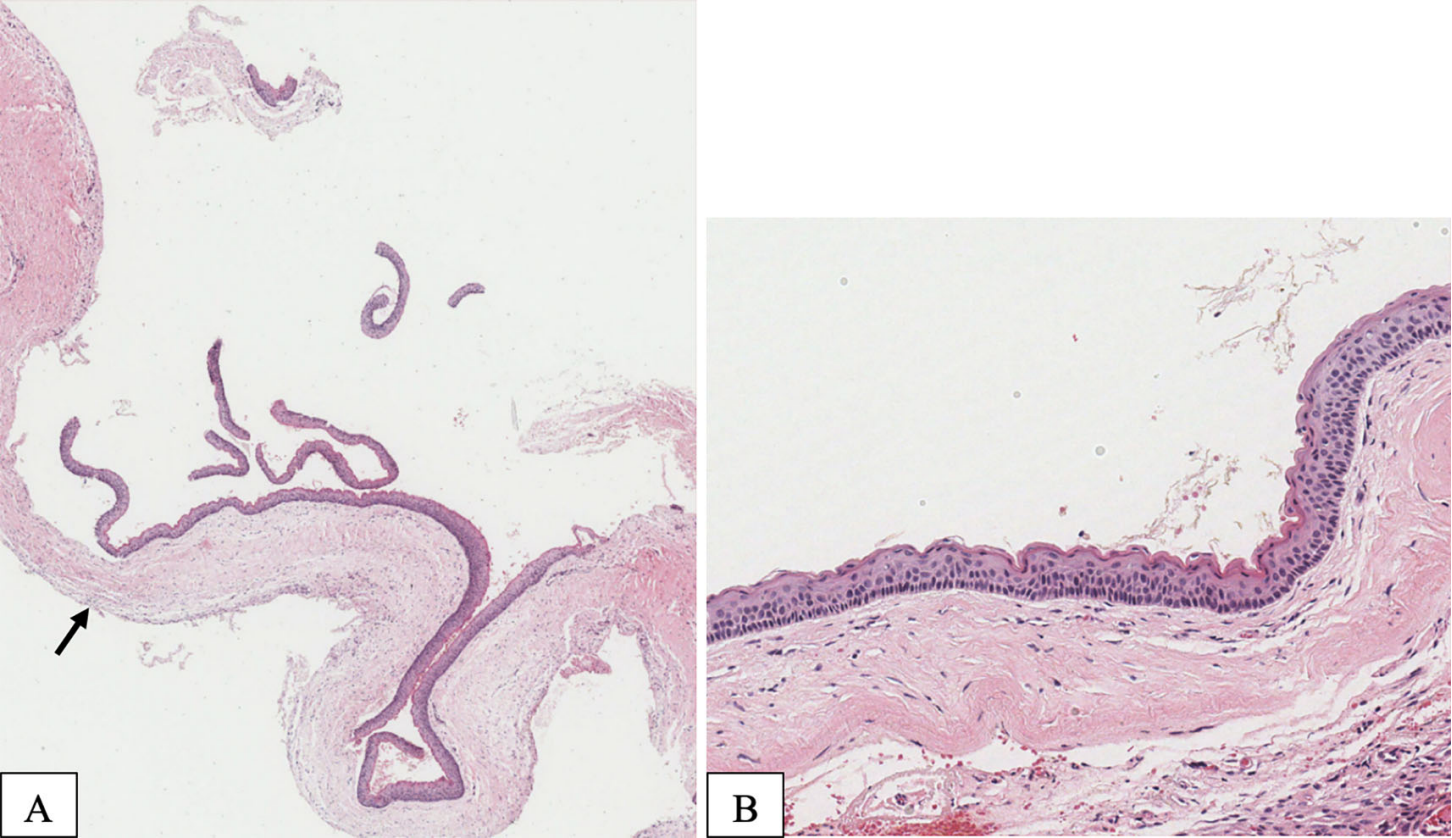
Case 1 histopathological examination. (A) Typical appearance of an OKC with a thin epithelial lining and a minimally inflamed fibrous capsule. The cyst is lined with a uniformly thin epithelium with epithelial detachment (arrow). (B) The lining epithelium is 5–10 cells thick and exhibits a parakeratotic surface layer with characteristic keratin corrugation and prominent basal cells with palisaded nuclei.

Aspiration of the lesion revealed cheesy material. The patient underwent surgery under general anesthesia for decompression using the novel tube technique, with an incisional biopsy and extraction of tooth 38. Histopathological analysis confirmed an OKC ( Figure 3).

**Figure 4.**
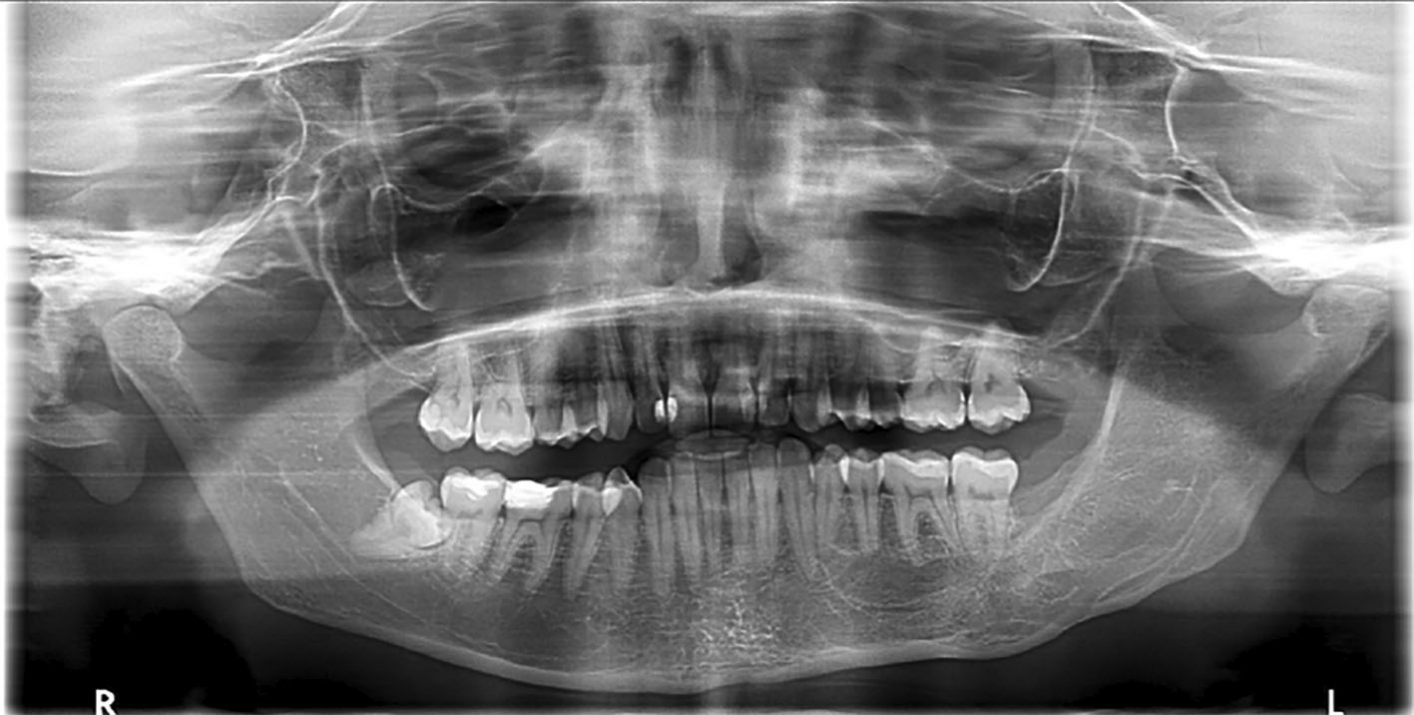
Case 1 post-operative OPG showing bony infill in the left mandible following cystic decompression.

The decompression tube remained in place for 14.5 months and was removed after radiographic evidence of healing ( Figure 4).

### Case 2

**Figure 5.**
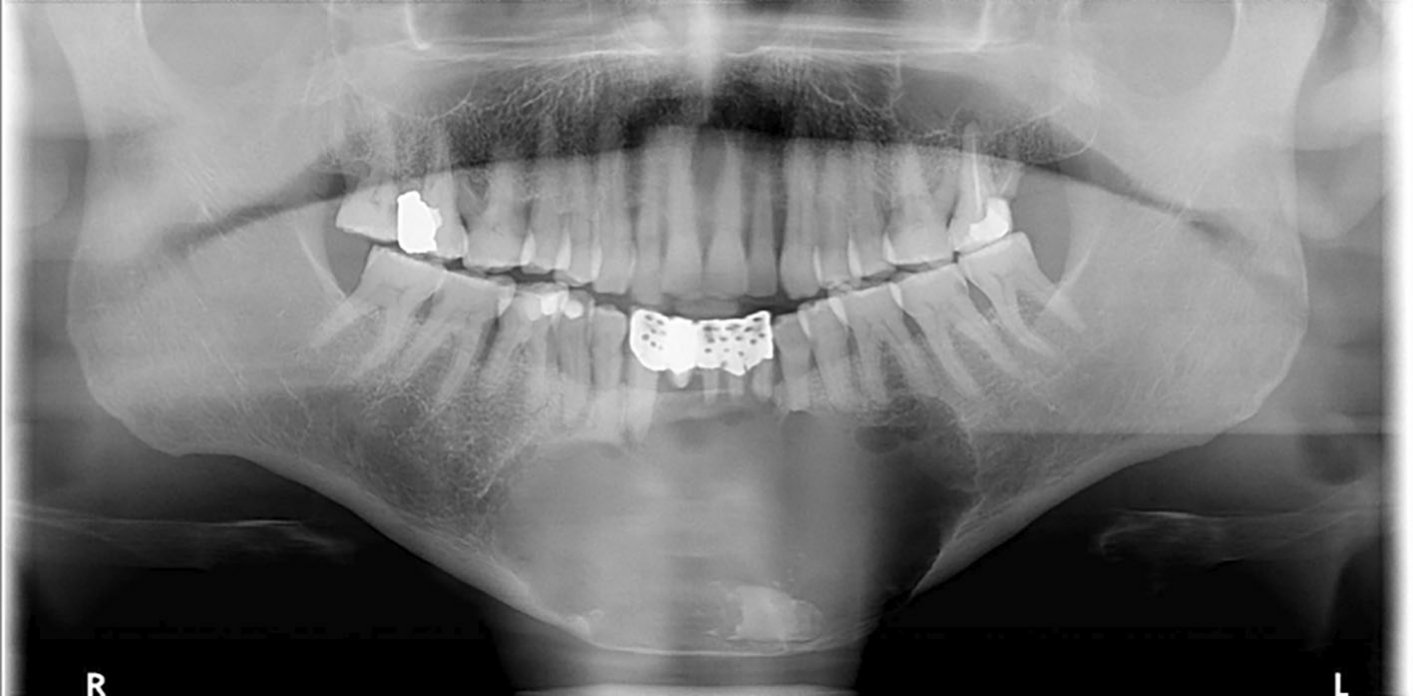
Case 2 pre-operative OPG, showing a large radiolucent lesion in the anterior mandible.

A 31-year-old male with diabetes who was receiving insulin injections visited our outpatient department. He did not have any allergies, and a visit to a private clinic incidentally revealed a large anterior mandibular lesion. He reported sudden asymptomatic swelling of his lower jaw that had begun three weeks prior. A physical examination revealed an anterior facial swelling that resulted in asymmetry and labial obliteration intraorally, along with noticeable crepitation upon palpation. There was no paresthesia, and the patient’s maximum mouth opening was normal. An OPG revealed a cystic lesion measuring 7.3 by 3.8 cm in the patient’s anterior mandible ( Figure 5).

**Figure 6.**
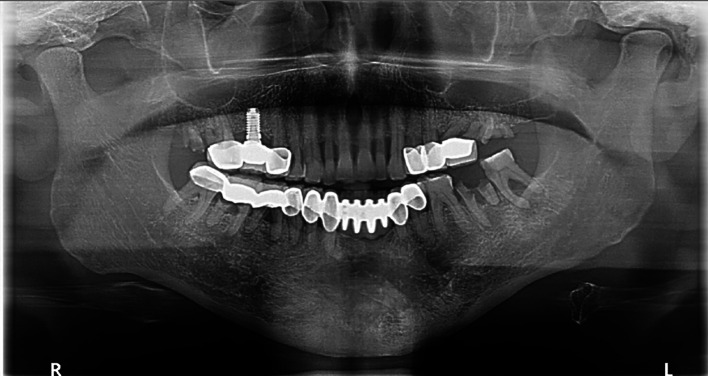
Case 2 OPG at 7-year follow-up showing complete bony infill in the anterior mandible following cystic decompression.

Aspiration revealed a cheesy-like material. The patient underwent decompression using the novel tube technique, along with an incisional biopsy and extraction of teeth 33, 32, and 31 under local anesthesia. A histopathological analysis confirmed an OKC. The decompression tube remained in place for 14 months and was removed following radiographic confirmation of healing. The patient was then kept on annual follow-up ( Figure 6).

### Case 3

**Figure 7.**
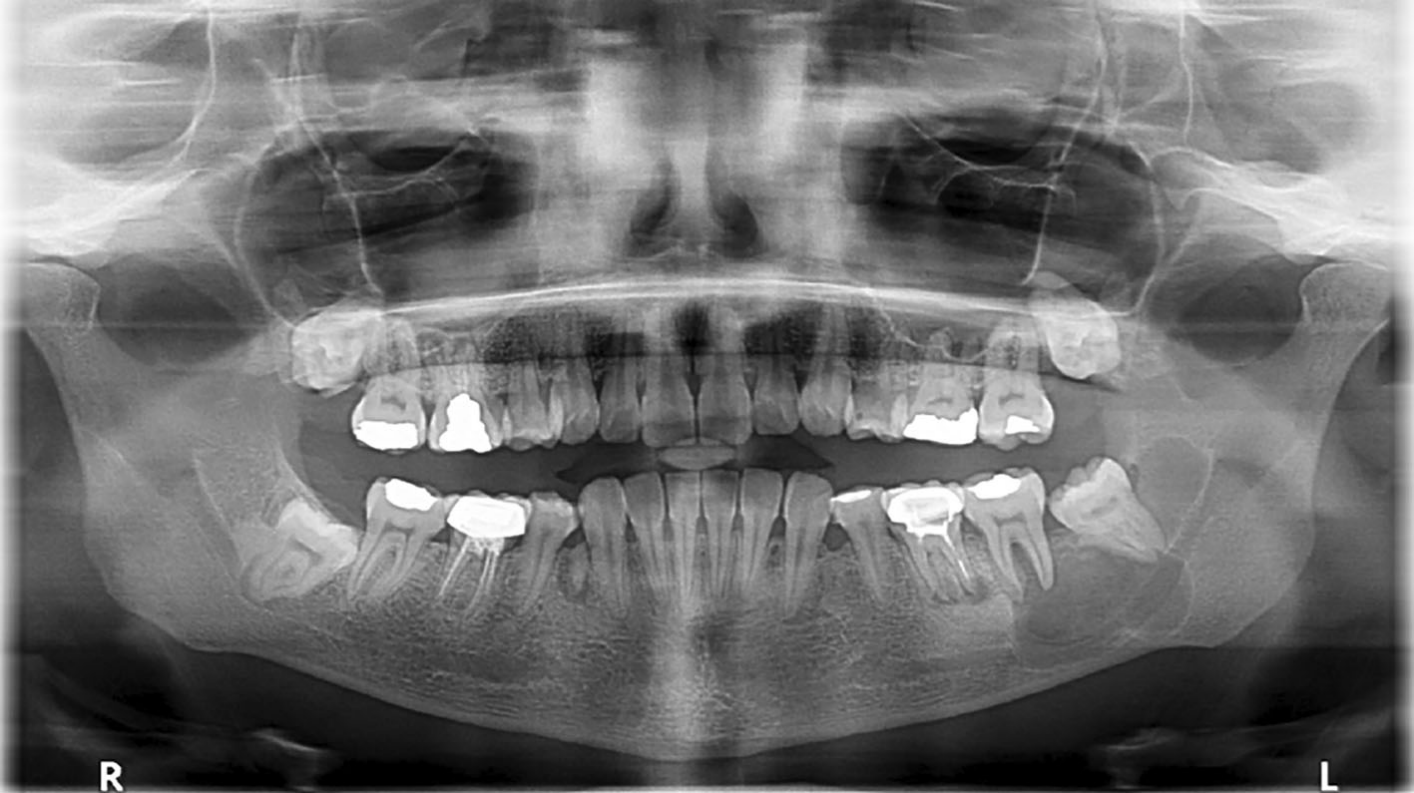
Case 3 pre-operative OPG, showing a large radiolucent lesion in the left mandible.

A 29-year-old female with no known medical conditions was referred to the OMFS clinic for evaluation of a cystic lesion on the left side of her mandible. The lesion was discovered incidentally two months prior during a routine dental visit. Upon examination, a left-sided facial swelling was noted. However, there were no signs of neurosensory deficits. Tooth 37 exhibited mobility, though no discharge was present, and the patient’s maximum mouth opening was within normal limits. A radiographic investigation revealed a multilocular radiolucency in the left mandible measuring 5.2 by 2.9 cm; it was associated with soft tissue impacting the left third molar ( Figure 7).

**Figure 8.**
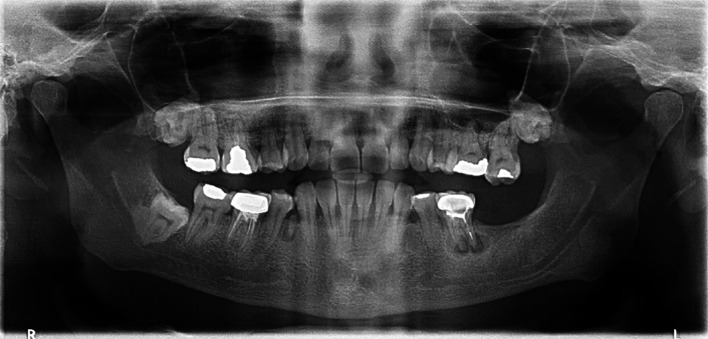
Case 3 post-operative OPG showing bony infill in the left mandible following cystic decompression.

A Cone Beam Computed Tomography (CBCT) scan confirmed the lesion’s extent, and aspiration revealed cheesy material. The patient underwent surgery under general anesthesia for decompression utilizing the novel tube technique, combined with an incisional biopsy and extraction of teeth 38 and 37. A histopathological analysis confirmed an OKC. The decompression tube remained in place for 8 months and was removed following radiographic confirmation of healing ( Figure 8).

### Case 4

**Figure 9.**
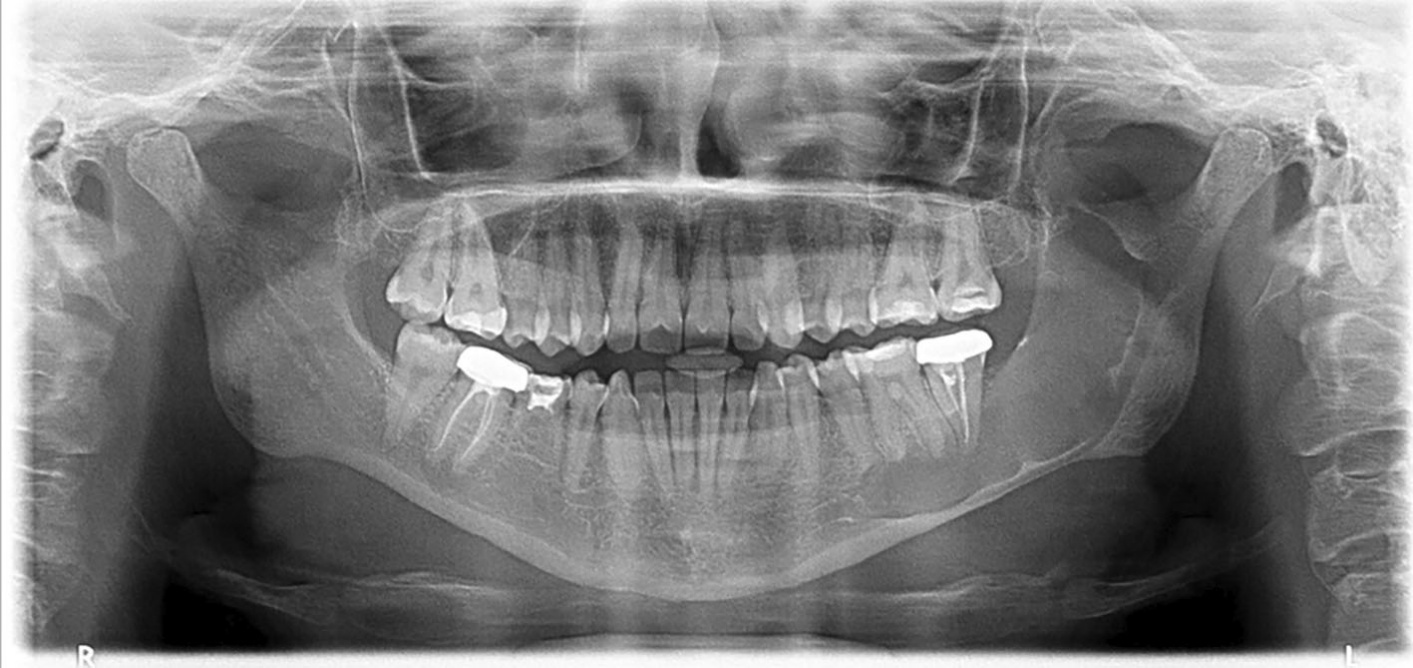
Case 4 pre-operative OPG, showing a large radiolucent lesion in the left mandible.

A 30-year-old female with a known history of hypothyroidism presented to the Emergency Department after being involved in a traffic accident. She was admitted to the neurosurgery department for management of a frontal sinus fracture. During her hospital stay, a CBCT scan of her facial bones incidentally revealed a mandibular lesion. An OPG revealed a multilocular radiolucent lesion measuring 5.6 by 1.7 cm in her posterior left mandible; it extended from the second premolar to the subcondylar region ( Figure 9).

**Figure 10.**
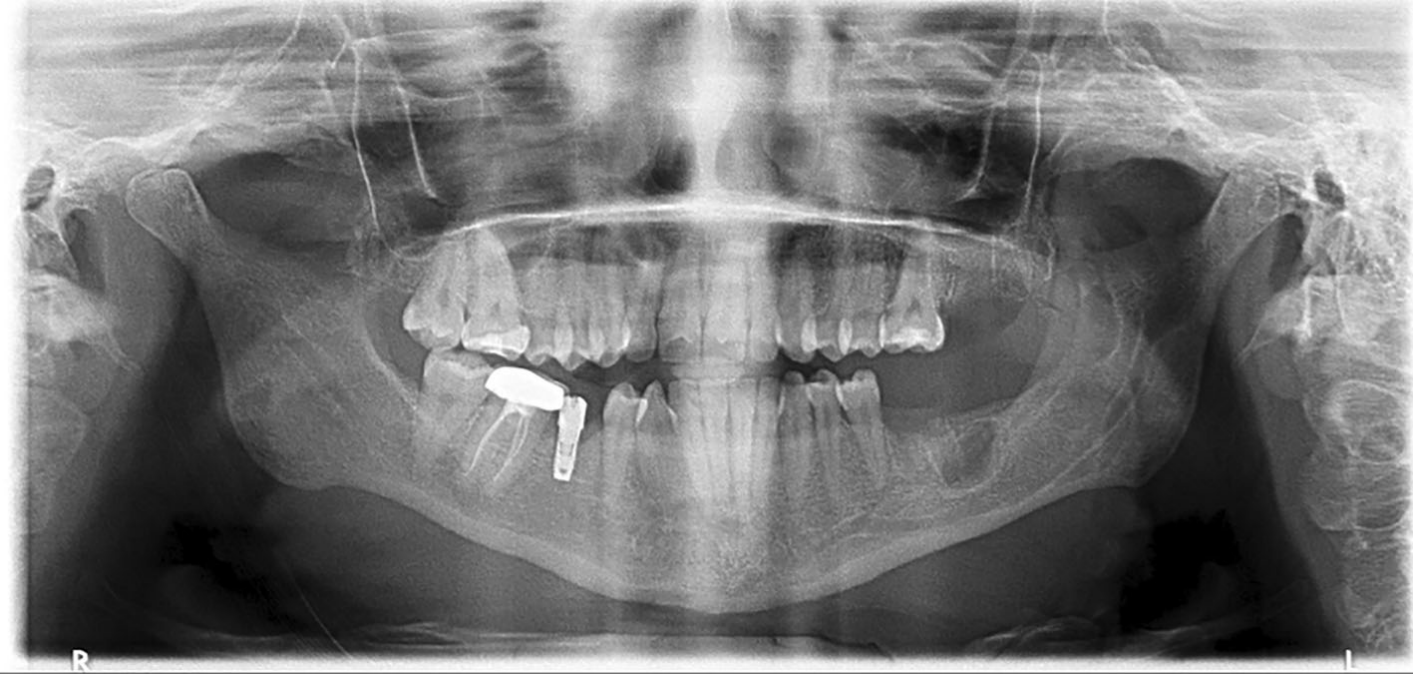
Case 4 post-operative OPG showing bony infill in the left mandible following cystic decompression and enucleation.

A CBCT scan confirmed the extent of the lesion. Aspiration yielded a cheesy-like material. The patient underwent surgical decompression utilizing the novel tube technique under general anesthesia, with an incisional biopsy and extraction of tooth 37. A histopathological examination confirmed an OKC. The tube remained in situ for 7 months and was removed after enucleation and extraction of tooth 36 ( Figure 10).

### Case 5

**Figure 11.**
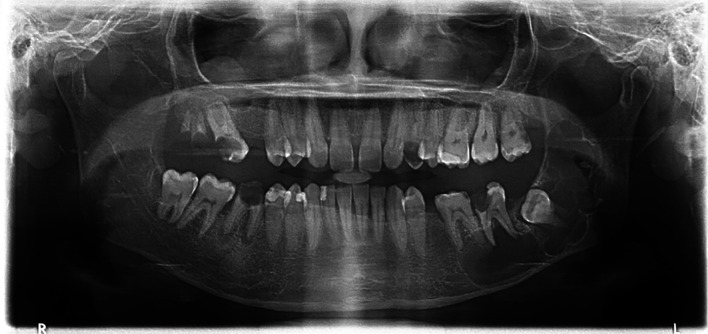
Case 5 pre-operative OPG, showing a large radiolucent lesion in the left mandible.

A 28-year-old female, with no known chronic medical conditions, presented to our clinic after an incidental finding of a large radiolucent lesion in her left mandible two months earlier at a private dental clinic. Upon examination, she exhibited no facial swelling or numbness, and an intraoral assessment revealed no buccal or lingual expansion. An OPG revealed a multilocular radiolucent lesion measuring 5.6 by 2.0 cm extending from the region of tooth 36 to the coronoid process ( Figure 11).

**Figure 12.**
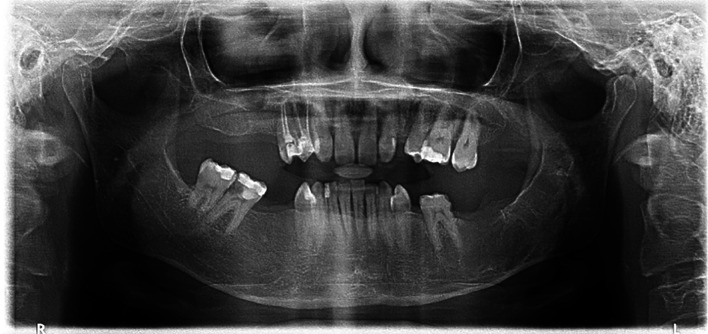
Case 5 post-operative OPG showing bony infill in the left mandible following cystic decompression and enucleation.

A CBCT scan revealed no evidence of bone perforation, and both the OPG and CBCT identified a pericoronal radiolucent lesion associated with a partially erupted tooth 48. Aspiration yielded a cheesy-like substance. The patient was taken to the operating room for decompression using the novel tube technique, followed by an incisional biopsy and extraction of tooth 38. Histopathology confirmed a diagnosis of OKC. The tube remained in place for 6 months and was subsequently removed after enucleation and extraction of tooth 37 ( Figure 12).

## Discussion

The term “odontogenic keratocyst” was first used by Maisonneuve in 1855, who described it as a “buttery cyst”. ^16^ However, the condition was officially termed “odontogenic keratocyst” by Philipsen in 1956. ^16^ It is derived from either the remaining dental lamina or the basal cells of the overlying epithelium. ^5^ An OKC can manifest as either a singular or multiple occurrence, and it is occasionally linked to a basal cell nevus syndrome, previously referred to as Gorlin or Gorlin-Goltz syndrome. ^16^ Therefore, it is essential to systematically investigate this syndrome when an OKC is detected in young individuals in order to identify and treat any underlying aggressive basal cell carcinoma or other tumor. ^16^

In 2005, following the publication of multiple case series that reported distinct features such as its aggressive behavior, gradual development, and substantial rate of recurrence, the World Health Organization (WHO) classified OKCs as benign odontogenic tumors and termed them “keratocyst odontogenic tumors.” Nevertheless, 12 years later, the aforementioned classification was reassessed and subsequently redefined as an odontogenic cystic lesion by the WHO. ^17^ Odontogenic keratocysts can be distinguished by the existence of a solitary or multiple-chambered radiolucent region that exhibits clearly defined boundaries and uniform margins. ^17^ Larger lesions frequently exhibit a Multilocular pattern. ^17^ Odontogenic keratocysts exhibit a comparatively elevated rate of recurrence within the first 5 years following treatment (i.e., 7–28%). ^17^

The therapeutic management of OKCs is widely recognized to be complex; surgical approaches need to be invasive and aggressive in order to prevent future recurrence. ^18^ Although there have been advancements in reconstructive surgery, the removal of a significant portion of the jaw still has a negative impact on quality of life and can lead to various complications. ^11, 19^ Conservative treatment can involve enucleation, curettage, marsupialization, and decompression. ^18^

Decompression of odontogenic cysts is a minimally invasive procedure that is even less invasive than marsupialization, enucleation, or curettage. It requires a smaller opening in the bone. ^20^ Decompression refers to any method used to alleviate the pressure within a cyst, which leads to its expansion. ^16^ The procedure involves creating an opening in the wall of the cyst. Commonly, decompression techniques employ a drain to ensure the cyst cavity remains open. ^21^ The earliest studies proposing decompression and marsupialization as therapeutic approaches for jaw cysts can be traced back to work by Partsch in 1892 and 1910. ^16^

There are no specific guidelines for the duration of the decompression or the required size reduction. However, previous research reported a 65% average decrease in size after maintenance for approximately 8 months. ^22^ Additionally, an 81% reduction was observed when decompression was maintained for an average of 17.5 months. ^22– 24^ In our first three case studies, the duration of decompression was extended to avoid recurrence; the latter two cases demonstrated that shorter periods of decompression were sufficient.

The relationship between patient age and the effects of decompression is a subject of debate. Some studies have found that younger patients experience greater reduction rates, while others have reported no correlation between patient age and the effectiveness of decompression. ^25, 26^ It is currently believed that patient age has no impact on the rate of reduction. Therefore, even in older individuals, the occurrence of postoperative complications can be minimized by reducing the size of large cystic lesions in the jaw via decompression. ^22^

Patients generally tolerate decompression well. However, the main disadvantage is the need for frequent follow-up. Effective case selection is crucial for the successful implementation of this technique. Patient adherence to follow-up cleaning is a crucial determinant of the efficacy of surgical decompression. The technique necessitates the patient’s cooperation and entails careful observation (i.e., multiple appointments) to monitor reductions in cyst size. Multiple patients reported experiencing discomfort or ulceration while the drainage tube was in place. In certain instances, it was necessary to readjust the position of the stent in order to enhance the comfort of the patient and avoid dislodgment.

## Conclusion

The Suhaim-Turayki Technique for treating OKCs using two drainage openings yielded excellent outcomes in our cohort of five patients. Complete bone regeneration was observed in a relatively short period of time, and there were no instances of recurrence during the extended follow-up period. For only two cases, recurrence was prevented by performing enucleation after significant size reduction of the cyst. This procedure can be used in cases involving other cysts or tumors where aggressive treatment is not preferred or feasible. Furthermore, the procedure is a viable option for primary management, provided that the histopathological diagnosis permits it.

## Ethical approval

Ethical approval was not required.

## Consent to publication

Written informed consent was obtained from the patients regarding the publication of their clinical details and clinical images.

## Data Availability

No data are associated with this article.

## References

[1] Tabrizi R Özkan BT Dehgani A : Marsupialization as a treatment option for the odontogenic keratocyst. *J. Craniofac. Surg.* 2012;23(5):e459–e461. 10.1097/SCS.0b013e31825b3308 22976707

[2] Thompson LDR : World Health Organization classification of tumours: pathology and genetics of head and neck tumours. *Ear Nose Throat J.* 2006;85(2):74. 10.1177/014556130608500201 16579185

[3] Hyun H-K Hong S-D Kim J-W : Recurrent keratocystic odontogenic tumor in the mandible: a case report and literature review. *Oral Surg. Oral Med. Oral Pathol. Oral Radiol. Endod.* 2009;108(2):e7–e10. 10.1016/j.tripleo.2009.04.030 19615649

[4] Shear M : Odontogenic keratocysts: clinical features. *Oral Maxillofac. Surg. Clin.* 2003;15(3):335–345. 10.1016/S1042-3699(03)00035-9 18088687

[5] Kitisubkanchana J Reduwan NH Poomsawat S : Odontogenic keratocyst and ameloblastoma: radiographic evaluation. *Oral Radiol.* 2021;37:55–65. 10.1007/s11282-020-00425-2 32030659

[6] Mallya S Lam E : *White and Pharoah’s Oral Radiology E-Book: Principles and Interpretation: Second South Asia Edition E-Book.* India: Elsevier;2019.

[7] Tabrizi R Kordkheili MRH Jafarian M : Decompression or marsupialization; which conservative treatment is associated with low recurrence rate in keratocystic odontogenic tumors? A systematic review. *J. Dent.* 2019;20(3):145–151. 10.30476/DENTJODS.2019.44899 31579687 PMC6732175

[8] Pogrel MA Jordan RCK : Marsupialization as a definitive treatment for the odontogenic keratocyst. *J. Oral Maxillofac. Surg.* 2004;62(6):651–655. 10.1016/j.joms.2003.08.029 15170272

[9] Wushou A Zhao Y-J Shao Z-M : Marsupialization is the optimal treatment approach for keratocystic odontogenic tumour. *J. Craniofac. Surg.* 2014;42(7):1540–1544. 10.1016/j.jcms.2014.04.027 24993466

[10] Kreppel M Zöller J : Ameloblastoma—Clinical, radiological, and therapeutic findings. *Oral Dis.* 2018;24(1-2):63–66. 10.1111/odi.12702 29480593

[11] Matsuda S Yoshimura H Yoshida H : Three-dimensional volumetric analysis of unicystic ameloblastoma before and after marsupialization using osirix software. *J. Hard Tissue Biol.* 2019;28(2):233–236. 10.2485/jhtb.28.233

[12] El-Hajj G Anneroth G : Odontogenic keratocysts—a retrospective clinical and histologic study. *Int. J. Oral Maxillofac. Surg.* 1996;25(2):124–129. 10.1016/S0901-5027(96)80057-9 8727585

[13] Pogrel MA : Treatment of keratocysts: the case for decompression and marsupialization. *J. Oral Maxillofac. Surg.* 2005;63(11):1667–1673. 10.1016/j.joms.2005.08.008 16243185

[14] Manor E Kachko L Puterman MB : Cystic lesions of the jaws-a clinicopathological study of 322 cases and review of the literature. *Int. J. Med. Sci.* 2012;9(1):20–26. 10.7150/ijms.9.20 22211085 PMC3222086

[15] De Castro MS Caixeta CA Carli ML de : Conservative surgical treatments for nonsyndromic odontogenic keratocysts: a systematic review and meta-analysis. *Clin. Oral Investig.* 2018;22:2089–2101. 10.1007/s00784-017-2315-8 29264656

[16] Muret M Malthiéry E Casenave T : Decompression: a first-intention treatment for “large” non-syndromic odontogenic keratocysts. *J. Oral Med. Oral Surg.* 2021;27(2):29. 10.1051/mbcb/2020063

[17] Vallejo-Rosero KA Camolesi GV Sá PLD de : Conservative management of odontogenic keratocyst with long-term 5-year follow-up: Case report and literature review. *Int. J. Surg. Case Rep.* 2020;66:8–15. 10.1016/j.ijscr.2019.11.023 31785568 PMC6889737

[18] Isolan C-P Moreira A-G Edges A : Successful conservative treatment of a mandibular unicystic ameloblastoma: 13-year follow-up. *J. Clin. Exp. Dent.* 2018;10(11):e1123–e1126. 10.4317/jced.54897 30607231 PMC6311408

[19] Ruihua L : Measures of Health-related Quality of Life in Huge Ameloblastoma Young Patients after Mandible Reconstruction with Free Fibula Flap. *J. Hard Tissue Biol.* 2014;23(2):261–266. 10.2485/jhtb.23.261

[20] Gao L Wang X-L Li S-M : Decompression as a treatment for odontogenic cystic lesions of the jaw. *J. Oral Maxillofac. Surg.* 2014;72(2):327–333. 10.1016/j.joms.2013.07.035 24071375

[21] Pogrel MA : Decompression and marsupialization as a treatment for the odontogenic keratocyst. *Oral Maxillofac. Surg. Clin.* 2003;15(3):415–427. 10.1016/S1042-3699(03)00038-4 18088693

[22] Lee S-T Kim S-G Moon S-Y : The effect of decompression as treatment of the cysts in the jaws: retrospective analysis. *J. Korean Assoc. Oral Maxillofac. Surg.* 2017;43(2):83–87. 10.5125/jkaoms.2017.43.2.83 28462191 PMC5410432

[23] Enislidis G Fock N Sulzbacher I : Conservative treatment of large cystic lesions of the mandible: a prospective study of the effect of decompression. *Br. J. Oral Maxillofac. Surg.* 2004;42(6):546–550. 10.1016/S0266-4356(04)00152-4 15544886

[24] August M Faquin WC Troulis MJ : Dedifferentiation of odontogenic keratocyst epithelium after cyst decompression. *J. Oral Maxillofac. Surg.* 2003;61(6):678–683. 10.1053/joms.2003.50137 12796876

[25] Anavi Y Gal G Miron H : Decompression of odontogenic cystic lesions: clinical long-term study of 73 cases. *Oral Surg. Oral Med. Oral Pathol. Oral Radiol. Endod.* 2011;112(2):164–169. 10.1016/j.tripleo.2010.09.069 21194990

[26] Rao S Rao S : Decompression as a treatment for odontogenic cystic lesions of the jaw. *J. Oral Maxillofac. Surg.* 2014;72(7):1231. 10.1016/j.joms.2014.03.035 24947962

