# Supplementary material for: Suhaim-Turayki Technique for the Decompression of Odontogenic Keratocysts: A Case Series
Source: F1000Res. 2025 May 20;14:504. [Version 1] doi: 10.12688/f1000research.163210.1 (PMC12397737; doi:10.12688/f1000research.163210.1)
Supplement: Peer review history

# Reviewer report: Reviewer response for version 1

**DOI:** 10.5256/f1000research.179519.r407589

**Published:** 2025-09-04

**Authors:** Ana Cláudia Garcia Rosa

**Affiliations:** 1 Federal University of Tocantins, Palmas, Brazil

This manuscript addresses a critical clinical problem: the management of odontogenic keratocysts (OKCs), which are characterized by their aggressive behavior and high recurrence rates. The authors present a modified decompression treatment approach using dual drainage openings and describe its application in five well-documented cases. The paper presents case descriptions and clinical and radiographic outcomes, suggesting that this technique is both feasible and effective. The writing is clear, and the topic is relevant for oral and maxillofacial surgeons, pathologists, and general practitioners.

 Despite these strengths, the manuscript would benefit from a clearer discussion of its limitations. The small cohort size reduces the generalizability of the findings, particularly considering the well-documented possibility of OKCs to recur even many years after treatment. Additionally, the discussion would be strengthened by highlighting the dependence on patient cooperation and the risks of treatment failure associated with poor compliance.

 In summary, the article makes a promising contribution by documenting a conservative approach with encouraging results. At the same time, the small sample size should temper the interpretation of the findings. Larger studies with long-term monitoring remain essential to confirm the durability of outcomes and to establish the factual recurrence rate.

Are enough details provided of any physical examination and diagnostic tests, treatment given and outcomes?

Yes

Is the background of the cases’ history and progression described in sufficient detail?

Yes

Is sufficient discussion included of the importance of the findings and their relevance to future understanding of disease processes, diagnosis or treatment?

Partly

Is the conclusion balanced and justified on the basis of the findings?

Yes

Reviewer Expertise:

Oral Pathology

I confirm that I have read this submission and believe that I have an appropriate level of expertise to confirm that it is of an acceptable scientific standard.

# Reviewer report: Reviewer response for version 1

**DOI:** 10.5256/f1000research.179519.r407588

**Published:** 2025-08-29

**Authors:** Afrah A. K. Aldelaimi

**Affiliations:** 1 University Of Anbar, Ramadi, Iraq

- The manuscript within the scope of the journal.
- The manuscript's quality and data presentation are acceptable and important for surgeons, pathologists, radiologist and general dental practitioner .
-  The manuscript advances our understanding of  jaw bone and odontogenic cysts of maxillofacial region .   
- The title should be rewritten to be more precise and explanatory.
-  Make the abstract more informative and it should represent the article's substance and be no more than 250 words long.
-  Include four to six keywords that are relevant to the manuscript but not stated in the title.

- Additional paragraphs to introduction about other  developmental cysts and tumors of maxillofacial region and its managements

 Suggested References:

 [Ref 1], [Ref 2] and [Ref 3] 

- Care should be taken to improve resolution and contrast for each figure in the manuscript .
- Authors should check for writing and typing errors.
- The statements in discussion are acceptable but few paragraphs about the justification of your findings and comparison with other recent relevant studies.

Only include current up to date references in the reference list and remove outdated ones.

Are enough details provided of any physical examination and diagnostic tests, treatment given and outcomes?

Yes

Is the background of the cases’ history and progression described in sufficient detail?

Yes

Is sufficient discussion included of the importance of the findings and their relevance to future understanding of disease processes, diagnosis or treatment?

No

Is the conclusion balanced and justified on the basis of the findings?

Yes

Reviewer Expertise:

Craniofacial surgery and maxillofacial surgery

I confirm that I have read this submission and believe that I have an appropriate level of expertise to confirm that it is of an acceptable scientific standard.

## References

[1] : Ectopic Upper Third Molar in Maxillary Sinus: A Case Report and Literature Review. *Indian Journal of Otolaryngology and Head & Neck Surgery* .2022;74(S3) : 10.1007/s12070-021-03039-0 4718-4721 10.1007/s12070-021-03039-0 36742931 PMC9895750

[2] : Ectopic upper third molar embedded in a dentigerous cyst of the maxillary sinus: a case report and literature review. *The Egyptian Journal of Otolaryngology* .2024;40(1) : 10.1186/s43163-024-00564-x 10.1186/s43163-024-00564-x

[3] : Management of a dentigerous cyst; a ten-year clinicopathological study. *BMC Oral Health* .2024;24(1) : 10.1186/s12903-024-04607-w 10.1186/s12903-024-04607-w 39044217 PMC11264404

# Reviewer report: Reviewer response for version 1

**DOI:** 10.5256/f1000research.179519.r390904

**Published:** 2025-08-13

**Authors:** Georges Aoun

**Affiliations:** 1 Lebanese University, Beirut, Lebanon

For me, this is a very well-written article about a very important oral pathology. The authors successfully described and treated the lesions.

 I have two minor comments: 1) It is essential to include a histopathological figure for all cases, similar to what was done in the first case. Additionally, please provide further details by adding arrows and more information regarding the histopathological confirmation of OKC in each case.

 2) Concerning the references, most of them are over five years old. Please consider adding or substituting them with more recent sources.

Are enough details provided of any physical examination and diagnostic tests, treatment given and outcomes?

Partly

Is the background of the cases’ history and progression described in sufficient detail?

Yes

Is sufficient discussion included of the importance of the findings and their relevance to future understanding of disease processes, diagnosis or treatment?

Yes

Is the conclusion balanced and justified on the basis of the findings?

Yes

Reviewer Expertise:

Oral medicine

I confirm that I have read this submission and believe that I have an appropriate level of expertise to confirm that it is of an acceptable scientific standard.
